# Changes in Working Conditions and Mental Health Among Intensive Care Physicians Across a Decade

**DOI:** 10.3389/fpsyt.2020.00145

**Published:** 2020-03-31

**Authors:** Petra Beschoner, Jörn von Wietersheim, Marc N. Jarczok, Maxi Braun, Carlos Schönfeldt-Lecuona, Lucia Jerg-Bretzke, Laurenz Steiner

**Affiliations:** ^1^Department of Psychosomatic Medicine and Psychotherapy, Ulm University Medical Center, Ulm, Germany; ^2^Clinic of Psychosomatics Kloster Dießen, Dießen am Ammersee, Germany; ^3^Department of Psychiatry and Psychotherapy III, Ulm University Medical Center, Ulm, Germany; ^4^III. Medical Clinic, University Medical Center Mannheim, Mannheim, Germany

**Keywords:** mental health, burnout, effort-reward-imbalance, intensive care physicians, working conditions, occupational stress

## Abstract

**Background:** International studies have shown that among physicians working in intensive care, a relatively high level of work load, an elevated risk of developing burnout and reduced mental health are frequent. The implementation of a legislative intervention in Germany with the goal to reduce the working hours of physicians, offered an opportunity to investigate the potential influence of occupational conditions on stress and mental health. The present study investigates working conditions, occupational stress and burnout risk in two samples of German Intensive Care Physicians in 2006 and 2016. The aim was to assess how occupational and private stress factors influenced burnout and Effort-Reward-Imbalance indices over this time-period.

**Methods:** Intensive care physicians were surveyed during the annual conference of their profession in two cross-sectional studies (10-year gap). Data on demographic (occupational, family), medical history, and mental health (burnout and Effort-Reward-Imbalance) were assessed by paper pencil questionnaires.

**Results:** In total, *N* = 2,085 physicians participated (2006: *N* = 1,403, 2016: *N* = 695), with *N* = 1,840 (2006 = 1,248; 2016 = 592) eligible for propensity score matching comparison. In general, more working hours per week and working days on weekends were associated with an increased effort/reward imbalance and higher burnout scores. From 2006 to 2016, reductions in working hours per week and days worked on weekends were accompanied by improvements in occupational stress (Effort-Reward-Imbalance) and by trend in mental health indices (burnout) after matching for differences in working conditions.

**Conclusions:** The study presents the changes concerning occupational stress factors and mental wellbeing in physicians working in intensive care in 2016 as compared to 2006. These findings may promote the implementation of preventive strategies in the vocational context to protect health and productivity of physicians, especially intensive care physicians.

## Introduction

The association of occupational stress and burnout with various types of health care professionals and the consequences for patients, employees, and institutions has gained increasing attention over the last two decades. Both stress and burnout are highly relevant topics across all medical fields and throughout all levels of hierarchy in medical institutions (1–3). This also applies to physicians, for whom high levels of stress and burnout are reported in the international literature (1, 4–7). Working in health care imposes various demands on physicians for various reasons: the challenging work environment, such as time constraints, scheduling of tasks and interruptions, lack of control over the workload, hierarchies, conflicts among colleagues and clashes with patients and their relatives are frequently mentioned challenges (8–10). Long working hours are another burden related to stress and burnout among physicians (11–13).

Particularly intensive care medicine, has been described as a health care section associated with high loads of stress (1, 14), and intensive care physicians appear to be severely affected by stress and burnout (5, 15). Specific stressors affecting physicians working in the field of intensive care are: time pressure, inadequate communication, lack of time management, traumatizing emergency situations with danger for body, and life of other persons and emotionally stressful “end-of-life-issues” (5, 16).

This professional stress plays an important role in the development of burnout in physicians (17). In a study on occupational stress, working conditions and private life among 1,202 physicians, Wu et al. (13) found that high extrinsic effort and more than 40 hours of work per week predicted high “Emotional Exhaustion,” one dimension of burnout. Tomioka et al. (18) found that in 702 of the physicians interviewed, long working hours were associated with an increased professional stress experience (Effort-Reward-Imbalance Questionnaire, ERI). However, they did not find a connection with depressiveness, which was also recorded. Martini et al. (19) substantiates these findings and assessed higher burnout rates among doctors with long working hours (>70 hours per week). They also examined the effects of the introduction of a working time limit on young physicians in 2006. Here, they found lower burnout levels, at least among young professionals, after the introduction of a working time limit. Shanafeldt et al. (20) were also able to demonstrate this positive effect of a reduction in working hours on the dimension “Emotional Exhaustion” of burnout (Maslach Burnout Inventory/ MBI-EE) in a large cohort of physicians in a longitudinal study.

In another longitudinal study with 143 physicians of internal medicine, Gopal et al. (21) found a positive effect of an arranged working time limitation on burnout, especially the dimension “Emotional Exhaustion.” Yet, in their systematic review, Ahmed et al. (22) conclude that reducing the working time of young doctors is not consistently accompanied by improvements in well-being and other factors need to be addressed.

However, the above presented findings on working hours, occupational stress and mental health in physicians come mainly from the USA and Asia. The extent to which they can be transferred to Europe and Germany in particular remains tentative.

Some data are now also available for Germany: Loerbrocks et al. (23) examined occupational stress in 416 German doctors according to the Effort-Reward-Imbalance model. They found a link between high values in the Effort-Reward-Ratio and reduced quality in patient care. Against the assumption, they could not present a significant correlation with depressiveness, which was found in a prospective study of 417 physicians in German hospitals, by Li et al. (24). They found that over time, increasing Effort-Reward-Imbalance values are predictive for the development of depression.

Voltmer et al. (25) were able to demonstrate an influence of occupational stress (Effort-Reward-Imbalance) and working hours on occupational satisfaction. They examined 414 privately employed physicians in Germany and found a significantly higher occupational stress burden compared to a sample of physicians in Norway. In a cross-sectional study, Ohlander et al. (26) compared a cohort of 85 physicians working in Sweden with a cohort of 561 physicians working in Germany in terms of working time and Effort-Reward-Imbalance. They were able to show that doctors in Germany have significantly longer working hours and higher Effort–Reward-Ratio Values. In addition, they were able to show a correlation between long working hours and the stressful occupational life.

These data show the importance of research on working conditions, occupational stress and possible health consequences in Europe. To our knowledge, in Europe no studies have so far been conducted in cohorts with more than 600 participants, examining working hours, stress and burnout in physicians. Furthermore, there is no study to date that has studied the effects of legal changes in working hours on stress and mental impairments in physicians after a period of 10 years.

In 2003, the Working Hours Act, which limits the working time of physicians by classifying on-call duty as working time, came into force in Europe. This Working Hours Act had to be implemented on January 1, 2007 (27). Thus, the aim of our study was on the one hand to record changes in working hours, occupational stress and burnout among physicians at a date before the implementation of the Working Hours Act (2006) and at a date 10 years later (2016). On the other hand, another aim of the study at hand was to record the relationships described in the literature (20–22) between the expected reduction in working hours and stress and burnout after a period of 10 years.

We assume that a reduction in working hours established by law has other effects on the experience of reward than a self-chosen reduction in working hours through part-time work (which is dearly paid). The associated positive effects have already been investigated (20). We expected a significant effect on the physicians' reward experience through the legally anchored work relief implemented by the employers.

In addition, we chose the period of a decade, to record effects resulting out of the long-term establishment and routine of the implementation of the Working Hours Act. Furthermore, we tried to avoid recording short-term effects, which might have occurred, for example, as a result of the knowledge of pending relief, or due to possible anger and uncertainty related to unstructured implementation of the changes, etc. We also wanted to avoid recording potential short-term effects due to the fact that the changes had not yet been implemented.

To assess possible changes across this decade we surveyed attendees of the annual meeting of the German Interdisciplinary Association for Intensive and Emergency medicine (DIVI) in 2006 and 2016 with the same methodology.

We chose the field of intensive care medicine since physicians in this field are particularly confronted with existential stress factors such as end-of-life-issues (5, 16), but not a specific large burden due to long working hours. Nevertheless, we expected a positive effect of the reduction of working hours on the experienced stress and burnout of intensive care physicians. This assumption is made on the basis of previous work (20, 21), suggesting that emotional distancing from emotionally stressful situation is enhanced by shorter working hours and, more free-time on the weekends.

In accordance with the literature above, we focused in especially on the interaction between working time, professional stress experience and the dimension “Emotional Exhaustion” in burnout. In the literature, working time is described as an influencing factor on stress experience (11) and the dimension “Emotional Exhaustion” of burnout syndrome (13) in physicians. We recorded working hours in the form of weekly working hours and the number of free weekends per month. To our knowledge, the latter has not yet been investigated and possibly influences the stress-related experience differently than the mere weekly working time. Simply put, a free weekend might offer different possibilities of regeneration than, for example, half hour less working time per day.

To measure stress, we used the Effort-Reward-Imbalance Questionnaire (28) because it is particularly suited to measure stress in workers who have many occupational interpersonal interactions (29). The ERI concept encompasses the development of occupational stress and the resulting health consequences. Numerous studies show its link to burnout and other adverse effects on health among health care professionals (30–32). In this context, serious effects such as suicidal tendencies, which are known to be an important issue among physicians (33) need to be addressed (33). Physicians in intensive care medicine have a particularly high suicide risk, compared to other specializations (34).

Burnout was recorded with the Maslach Burnout Inventory (35) and again, we put the focus on the dimension “Emotional Exhaustion.” This affective component of the Maslach burnout construct is specifically influenced by Effort-Reward-Imbalance and impacts the quality of patient care (36). Improvements in the mentioned indicators would provide evidence consistent with a current model of the effects of working conditions on burnout and Effort-Reward Imbalance (28, 37).

Aim of this study was to assess changes between 2006 and 2016 in the private and professional situation of physicians working in intensive care units. Firstly, we expected changes in the working hours, as a result of the Working Hours Act. Secondly, potential differences in stress experience and mental health indices were measured. Thirdly, links between private and professional factors as well as occupational stress (ERI) and burnout (MBI-EE) were assessed, with an expected positive effect of weekly working hours and free weekends on ERI and MBI-EE. Additionally, the question if effects of working time on MBI-EE were mediated by ERI, was addressed. Finally, we were interested to see if a possible mediation between work hours and free weekends with Effort-Reward-Imbalance and “Emotional Exhaustion” could be found. These analyses were conducted on the data of 2006 and 2016 to assess the stability of these correlations.

## Methods

We surveyed intensive care physicians at the annual meeting of the German Interdisciplinary Association for Intensive and Emergency Medicine (Deutsche Interdisziplinäre Vereinigung für Intensiv- und Notfallmedizin, DIVI) in 2006 and 2016. About 6,000 attendees from German speaking countries registered for this conference in 2016 (38).

The data collection procedure in 2006 and 2016 was identical. A stand with questionnaires and a lockable return box was set up in the foyer area of the DIVI conference. Two posters were placed on a partition wall, referring to the survey carried out during the conference and our stand. A contact person was present at all times to acquire participants and answer any related questions. In addition, questionnaires were handed out personally to the participants in the entire conference area. The participants received the questionnaire with a short explanation of its contents as well as the anonymity and the possibility of sending the questionnaire by post. The participants were informed on the cover page of the questionnaire that they agreed to participate by filling in the form and submitting it anonymously to the collection box.

The data collected included personal information, information about the family, the professional situation and the medical history using an identical questionnaire in 2006 and 2016. In order to assure that the questionnaire was filled out by an intensive care physician despite the survey taking place at an intensive care medical congress, we recorded the medical field of activity in our questionnaire. The section on medical history covered the entire life span. To assess burnout, ERI and Overcommitment, standardized instruments were used (32, 35). The duration to complete the questionnaires was about 15-20 min and they were returned anonymously. A total of *N* = 2,551 Questionnaires (in 2006) and *N* = 1,627 Questionnaires (in 2016) were handed out and *N* = 1,403 (in 2006) and *N* = 695 (in 2016) questionnaires were returned. The return rate was 55% in 2006 and 42% in 2016. Questionnaires with too much missing data were excluded. Thus, in 2006 *N* = 1.248 and in 2016 *N* = 592 data sets were analyzed. Data sets of participants with missing information on sex and age were completely discarded. When data of items in the standardized instruments (ERI, OC, BDI-II, MBI) were missing, these questionnaires were excluded from the evaluations (listwise deletion). Missing data on other items was coded as “not reported.”

The Ethics Commission of the University of Ulm approved the study design (192/16).

Some of the data will simultaneously be published in a dissertation.

### Psychometric Tests

The following psychometric research instruments were used.

#### Maslach Burnout Inventory D (MBI-D) (35, 39, 40)

The original MBI by Maslach and Jackson, was chosen in the German, reviewed and revised version by Büssing and Perrar (MBI-D).

Even though at least four German translations exist, the version by Büssing and Perrar is the only one authorized by Maslach (41). Since the 1992 version of the translated instrument has been revised and reviewed but not yet published, the approval was given by Prof. J. Glaser of the University of Innsbruck.

The MBI-D consists of 21 items. Subjects are asked to rate each item, ranging from 1 (never) to 6 (very often). The scale itself has three domains: “Emotional Exhaustion” (MBI-EE) (9 items), “Depersonalization” (MBI-DP) (5 items) and “Reduced Personal Accomplishment” (MBI-PA) (7 items). “Emotional Exhaustion” means feeling emotionally overstrained by work. Depersonalization reflects an indifferent or cynical attitude toward patients. Personal Accomplishment addresses feelings of competence and achievement at work.

The MBI provides semi-continuous outcome variables (item mean, sum score) that allow the scientific consideration of the burnout construct in comparison to other constructs such as ERI and the development of burnout severity over time. We used the item means, since this approach methodically meets the problem of missing items. We also calculated sum scores to make for enhanced comparability with data from other publications.

A categorical consideration of the burnout construct measured with the MBI-D is possible, but difficult due to the lack of reliable and stable cut-off values. Nevertheless, a categorical analysis of the burnout values allows a rough assessment of the prevalence, which is sometimes of assistance and necessary, especially in the medical context. For this interpretation we defined a cut-off of >4.5 points (item mean) for the domain “Emotional Exhaustion” (oral communication in 2006 and 2016 with Prof. J. Glaser, University of Innsbruck). We focused solely on “Emotional Exhaustion” based on the early theory of Maslach et al. (42) where “Emotional Exhaustion” is the core element of burnout, and is the most obvious manifestation of this complex construct. In addition, Wang et al. (43) show that the dimension “Emotional Exhaustion” in particular is significantly related to long working hours, high job effort and low reward. It has also been shown that “Emotional Exhaustion” is particularly influenced by ERI with effects on the quality of care [e.g., Weigl et al. (36)].

#### Effort-Reward-Imbalance (ERI) (32, 37, 44)

Effort is measured by 5 items. All questions refer to one's present occupation and subjects are asked to indicate in how far the items reflect their typical work situation (Sample question Effort: “Due to the high workload, there is often a great deal of time pressure.” Sample question Reward: “I get the recognition I deserve from my superiors”). Higher ratings point to higher efforts, ranging from 1 (does not apply) to 5 (does apply). The sum of these ratings is computed as a measure of extrinsic effort (range from 5 to 25). Reward is measured by 11 items. After variable recoding procedures, lower ratings point to lower rewards, with the sum ranging from 11 to 55. The ERI ratio is computed as ratio of the effort score divided by the reward score and then multiplied by a correction factor to correct the difference in the numbers of items of the two scales. Values > 1 indicate a negative imbalance (ERI) between effort and reward (32, 44). Finally, overcommitment (OC) is measured by 6 items ranging from 1 (low) to 4 (high). (Sample item Overcommitment: People close to me say I sacrifice too much for my job). Sum scores for overcommitment range from 6 to 24. A score > 16 indicates that a subject is at risk to develop stress symptoms (32).

### Statistical Analysis

All statistical computations were performed with the Statistical Package for the Social Sciences (SPSS), Version 24.0 and Stata 15.1 SE (Stata Corp, USA).

#### Bivariate Analysis

In a first step, significance tests were performed on all categorical variables using Chi square tests to estimate average, unadjusted between year differences. Continuous variables were tested on skewness and kurtosis for normal (Gaussian) distribution (using Stata's *ladder* command) and linearly transformed according to the ladder of power (45). Yet, none of the tested variables were eligible for linear transformation. Subsequently Mann-Whitney *U*-Test for nonparametric data was applied. Statistical significance was assumed for *p* ≤ 0.05.

#### Average Treatment Effect Estimation Using Propensity Score Matching

To improve comparability of the samples in 2006 and 2016 and to reduce the risk of bias due to confounding covariates, a statistical matching technique (i.e., propensity score matching) was applied (Cf. Table 4). This method is superior when comparing treated (or exposed) vs. untreated (or unexposed) populations from observational, non-experimental data (45). Here, similar observations are compared on the outcome variable of interest using propensity scores from a set of matching variables, which can be assumed to have an influence on working time and occupational stress. These variables were: sex, age, working full-time/part-time, working in hospital/private practice, leadership/assistance position. On all categorical matching variables, missing information was recoded into a new missing category prior to effect estimations (i.e., sex: male, female, missing) to include as much information as possible into the model (46). The Stata command “*teffects psmatch”* was used to estimate the average treatment effect from the present observational.

#### Mediation Analysis

Four distinct mediation models were estimated (Cf. **Figure 2**) [per year for the X variables “total working time (hours per week) and number of free weekends (per month)] using a structural equation approach as suggested by Ditlevsen et al. (47). The purpose of the mediation analysis is to see if the underlying association between worktime and free weekends and MBI is mediated by ERI in the same manner in both years. One of the main advantages of the structural equation approach is a simultaneous estimation of all specified pathways. Following the procedure for mediation analysis suggested by Hayes et al. (48) our equations were identified to estimate the direct, indirect, and total effect (see **Figure 2** for a graphical representation of the model). All mediation models were adjusted for the potential differences across the two sampling timepoints using age (years), sex (male vs. female), and position (Managing position yes vs. no). The indirect and total effects are reported in **Figure 2**. The model parameters were estimated using a maximum likelihood procedure allowing missing values (Expectation-Maximization) to maintain as much information as possible. To better adjust for between subject variations, an alternative robust variance estimation method (bootstrapping with 1,000 replications) was performed.

The reported regression coefficients were not standardized to keep the original metric of X and Y for two reasons. First, to allow meaningful interpretation of the indirect effect and second, to allow for between year comparisons of the regression coefficients. For example, an indirect effect of “Work hours (per week)” (X) on MBI-EE (Y) can be interpreted as the difference in MBI-EE unit (Score change) attributional to the indirect pathway through the mediator ERI [see Ditlivsen et al. (47) for an in-depth discussion].

## Results

### Descriptive Data Analysis of the Samples in 2006 and 2016

A total of *N* = 1,385 participants were available in 2006 and *N* = 687 in 2016. In 2006 we excluded *N* = 455 data sets, in 2016 *N* = 95 because the questionnaires were too incomplete. The total analysis sample comprise *N* = 1,840 participants. In 2006 38.8% of the participants were female. This proportion slightly increased in 2016 (43.2%). The mean age increased significantly by 3.5 years to 44.8 years (Cf. Table 1).

Table 1Comparison of family, occupational and health characteristics after matching.

**Table:** 

		**2006**	**2016**	***P***	***V***
		***N* (%)**	***N* (%)**		
**Gender**					
Female		485 (38.86)	256 (43.24)	0.073	−0.0417
Male		763 (61.14)	336 (56.76)		
**Partnership**					
No		183 (14.66)	95 (16.05)	<0.001	0.1812
Yes		1.052 (84.29)	451 (76.18)		
Not reported		13 (1.04)	46 (7.77)		
**Children**					
No		513 (41.11)	235 (39.70)	0.643	0.0219
Yes		720 (57.69)	352 (59.46)		
Not reported		15 (1.20)	5 (0.84)		
**Full-time contract**					
No		168 (13.46)	136 (22.97)	<0.001	0.0219
Yes		1.067 (85.50)	455 (76.86)		
Not reported		13 (1.04)	1 (0.17)		
**Main occupation**					
Private Practice		167 (13.38)	44 (7.43)	<0.001	0.982
Hospital		1.051 (84.21)	541 (91.39)		
Not reported		30 (2.40)	7 (1.18)		
**Working position**					
Senior position		485 (38.86)	330 (55.74)	<0.001	0.1601
Assistance position		733 (58.73)	255 (43.07)		
Not reported		30 (2.40)	7 (1.18)		
**Acute/chronic illness**					
No		918 (73.56)	400 (67.57)	0.008	0.0621
Yes		330 (26.44)	192 (32.43)		
**Medication intake**					
No		1.016 (81.41)	426 (71.96)	<0.001	0.1072
Yes		232 (18.59)	166 (28.04)		
**Antidepressant intake**					
No		1.235 (98.96)	585 (98.82)	0.786	0.0063
Yes		13 (1.04)	7 (1.18)		
**Sedative intake**					
No		1.229 (98.48)	582 (98.31)	0.788	0.0063
Yes		19 (1.52)	10 (1.69)		
**Analgesic intake**					
No		1.117 (89.50)	524 (88.51)	0.523	0.0149
Yes		131 (10.50)	68n (11.49)		
**Work missed due to overload**					
No		1.177 (94.31)	551 (93.07)	0.300	0.0242
Yes		71 (5.69)	41 (6.93)		
**Currently in psychotherapy**					
No		1.213 (97.20)	578 (97.64)	0.584	−0.0128
Yes		35 (2.80)	14 (2.36)		
**Previously in psychotherapy**					
No		1.067 (85.50)	512 (86.49)	0.570	−0.0133
Yes		181 (14.50)	80 (13.51)		
**Depression in medical history**					
No		990 (79.33)	501 (84.63)	0.007	−0.0632
Yes		258 (20.67)	91 (15.37)		
**Of which diagnosed by a specialist**	No	189 (74.41)	56 (63.64)	0.053	0.1045
	Yes	65 (25.59)	32 (36.36)		
**Suicide attempt**					
No		1.237 (99.12)	589 (99.49)	0.063	−0.0409
Yes		11 (0.88)	3 (0.51)

**Table:** 

		**2006**			**2016**			***P***	***R*** **95% conf. interval**
		***N***	***M***	***SD***	***N***	***M***	***SD***		
Age		1.248	41.3	7.83	592	44.8	9.38	<0.001	−0.19 (−0.23 to −0.15)
Number of children (when yes)		605	1.92	0.844	334	1.63	1.03	<0.001	0.15 (0.09 to 0.21)
Work hours (per week)		1.248	55.7	13.5	592	53.2	12.2	<0.001	0.09 (0.04 to 0.13)
Free weekends (per month)		1.248	1.87	0.732	592	2.11	0.759	<0.001	−0.15 (−0.20 to −0.11)

*P-values from chi square test and for continuous data from Mann-Whitney-U-Test. Effect size (66): Cramers V = 0.1 small effect, V = 0.3 medium effect, V = 0.5 large effect*.

#### Family Situation

In 2006, fewer physicians lived without a partner and less had children than in 2016. The number of children living in the household of the participants at the time of the survey decreased slightly from 2006 to 2016. (Cf. Table 1).

#### Professional Situation

The percentage of surveyed intensive care physicians working full-time at the time of the survey, decreased significantly by around 9 percentage points from 2006 to 2016. The percentage of those working in a hospital increased significantly by around 7 percentage points in this decade, while the percentage points of those working in private practices decreased. And in 2016 nearly 17 percentage points more of physicians surveyed held a senior position than in 2006 (Cf. Table 1).

### Changes in Worktime, Burnout, and Mental Health Between the Samples in 2006 and 2016

#### Worktime

The number of free weekends (without being on call) increased significantly from a mean of 1.9 in 2006 to a mean of 2.1 in 2016. Regarding the number of hours worked per week, those surveyed listed approximately 3 hours more in 2006 than in 2016. (Cf. Table 1). Similar to these results, the working time of full-time employees decreased by 2.5 hours from 2006 to 2016 (95% CI −3.78 to −1.24, *p* ≤ 0.001, Mann-Whitney *U*-Test). This shows that the reduction in working hours from 2006 to 2016 is not the sole effect of more part-time work or a higher proportion of women.

Despite this improvement, there was no significant difference between 2006 and 2016 in the frequency with which the question “*whether the participant had ever been on sick leave due to overload*” was answered with yes. (Cf. Table 1).

#### Health and Medical History

There was no significant difference in participants suffering from an acute or chronic disease between 2006 and 2016. The frequency of medication intake, especially antidepressant intake, sedative intake, and analgesic intake also did not differ significantly between 2006 and 2016. A depression in the medical history was found by more than 5% participants less in 2016 than in 2006. The percentage of those who had the diagnosis confirmed by a medical specialist, however, had increased significantly by nearly 11 percentage points. This effect did not turn out to be significant. The number of respondents currently undergoing psychotherapeutic treatment did not change significantly between 2006 and 2016. Similarly, the number of those who had previously undergone psychotherapeutic therapy remained unchanged. Suicide attempts were less frequent in 2016 compared to 2006, but due to the small number of reported cases no statements can be made about the significance level.

#### Current Burnout Symptoms

The continuous variables (item means) in the domains “Emotional Exhaustion” “Personal Accomplishment” of the Maslach Burnout Inventory show a significant improvement from 2006 to 2016. In the domain “Depersonalization,” the item means did not differ significantly from 2006 to 2016 (Cf. Table 2). The sum scores show the same trend.

**Table 2:** Comparison of questionnaire scores of the study samples in 2006 and 2016.

**Questionnaire scores**	**2006 (*****N*** **=** **1.248)**		**2016 (*****N*** **=** **592)**			**Cohens D 95% conf. interval**
**MBI (item means)**	***M***	***SD***	***M***	***SD***	***p***	
Emotional Exhaustion	3.17	0.95	3.0	0.99	<0.001	0.18 (0.08 to 0.28)
Depersonalization	2.55	0.90	2.48	0.90	0.083	0.08 (−0.01 to 0.18)
Personal Accomplishment	4.6	0.65	4.74	0.60	<0.001	−0.22 (−0.32 to −0.12)
**MBI (sum scores)**						
Emotional Exhaustion	28.5	8.54	27.0	8.91	<0.001	0.18 (0.08 to 0.28)
Depersonalization	12.8	4.44	12.4	4.48	0.083	0.19 (0.09 to 0.29)
Personal Accomplishment	32.2	4.54	33.2	4.19	<0.001	0.08 (−0.01 to 0.18)
**ERI**						
Effort	15.2	3.34	14.4	3.52	<0.001	0.24 (0.14 to 0.34)
Reward	41.9	7.91	46	7.43	<0.001	−0.53 (−0.63 to −0.43)
Esteem	19.6	4.17	20.9	4.09	<0.001	−0.30 (−0.4 to −0.2)
Job security	7.97	1.88	8.61	1.64	<0.001	−0.35 (−0.45 to −0.25)
Job promotion	14.3	3.8	16.6	3.5	<0.001	−0.60 (−0.70 to −0.50)
Effort-Reward-Ratio	0.85	0.35	0.73	0.29	<0.001	0.38 (0.28 to 0.48)
Overcommitment	14.2	3.82	13.9	3.7	0.122	0.08 (−0.02 to 0.18)

*Sample Size N = 1,840. P-Values from Mann-Whitney-U-Test. Effect size (66): Cohens D, D = 0.1 small effect, D = 0.3 medium effect, D = 0.5 large effect*.

**Figure 1:**
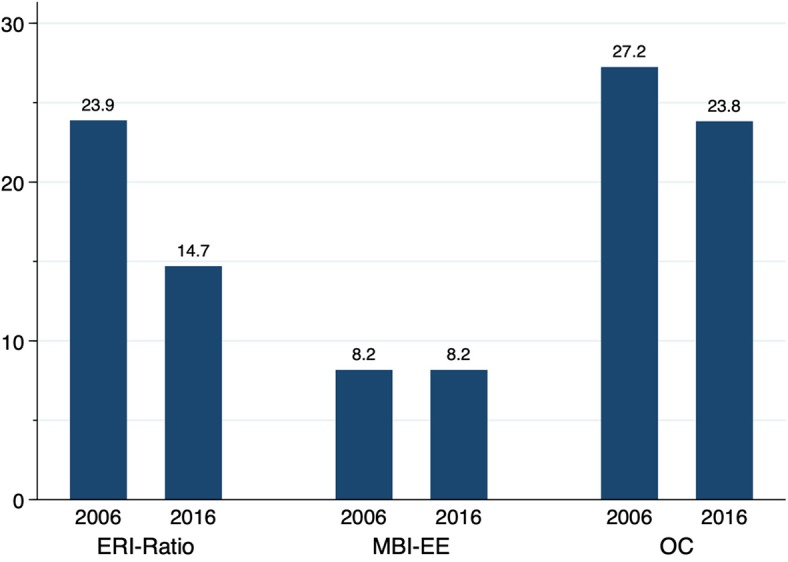
Descriptives of burnout risk, effort-reward-imbalance and overcommitment (percentage). *P*-values derived from propensity score matched data (ATE). Effort-Reward-Ratio >1 (*p* ≤ 0.001), MBI-EE score > 4.5 (*p* = 0.37), OC score > 16 (*p* = 0.15). Effect size: Cramers *V* = 0.1 small effect, *V* = 0.3 medium effect, *V* = 0.5 large effect (66): ERI-Ratio: −0.11; MBI-EE: −0.00; OC: −0.04.

The dichotomous analysis of the MBI data indicate a similar proportion of 8.2% (*N* = 102 in 2006; *N* = 48 in 2016) reaching the 4.5 cutoff for “Emotional Exhaustion” (*p* = 0.37; Cf. Figure 1).

#### Effort-Reward-Imbalance

An Effort-Reward-Imbalance (values > 1) existed in 2006 for 23.9% (*N* = 298) of the participants; in 2016, the percentage was about 10 percentage points lower (14.7%; *N* = 87) (*p* ≤ 0.001). The percentage for Overcommitment did not differ significantly from 2006 to 2016 (2006: 27.2%, *N* = 340) vs. 2016: 23.8%, *N* = 48; *p* < 0.15) (Cf. Figure 1).

#### Correlations

Table 3 reports Pearson's correlations between socioeconomic/professional factors and assessment of Effort-Reward-Imbalance and burnout, excluding the “not reported” categories. The more hours worked per week and less free weekends per month showed a statistically significant correlation with higher scores for Overcommitment, Effort-Reward-Imbalance and “Emotional Exhaustion” (MBI-EE). Having children (No/Yes) correlates significantly with slightly higher scores on ERI, while having a partner (No/Yes) correlates statistically significant and positively with MBI-EE and statistically significant and negatively with Overcommitment. Men seem to report lower MBI-EE scores compared to women. Working in an assistance position correlates statistically significant with lower Overcommitment and higher MBI-EE.

**Table 3:** Results of the correlation analyses (combined 2006 and 2016).

	**ERI**	**OC**	**MBI-EE**
Gender (W vs. M)	0.018	−0.032	−0.083^**^
Working position (Senior vs. Assistance; *N* = 1,803)	−0.019	−0.064^**^	0.091^**^
Work hours (per week)	0.200^**^	0.170^**^	0.066^**^
Free weekends (per month)	−0.179^**^	−0.125^**^	−0.113^**^
Children (*N* = 939)	0.074^*^	0.028	0.034
Partnership (*N* = 1,781)	−0.015	−0.058^*^	0.098^*^

*M, men; W, women; OC, Overcommitment; ERI, Effort-Reward Ratio; MBI-EE, Emotional Exhaustion item scores. Correlation coefficient [Pearsons R; effect size: R = 0.1 small effect, R = 0.3 medium effect, R = 0.5 large effect; Cohen (66)]*,

* *p ≤ 0.05*,

** *p < 0.01. N = 1,840 unless indicated otherwise*.

**Table 4:** Average treatment (year) effect from observational data using logistic models after propensity-score matching (2016 vs. 2006), caliper 0.15.

***N* = 1,840**	**ATE Coef**.	**AI Robust std. err**.	**z**	**P>|z|**	**95% conf. interval**	
Work hours (per week)	−1.94	0.63	−3.07	<0.01	−3.18	−0.70
Free weekends (per month)	0.18	0.04	4.41	<0.01	0.10	0.26
ERI Effort	−0.82	0.19	−4.22	<0.01	−1.20	−0.44
ERI Reward	3.46	0.41	8.36	<0.01	2.65	4.28
ERI Esteem	1.10	0.23	4.83	<0.01	0.65	1.54
ERI Job security	0.68	0.10	6.93	<0.01	0.49	0.88
ERI Job promotion	1.69	0.19	8.74	<0.01	1.31	2.07
Effort-Reward-Ratio	−0.11	0.02	−7.05	<0.01	−0.15	−0.08
Overcommitment	−0.23	0.21	−1.09	0.27	−0.64	0.18
ERI categorical	−0.08	0.02	−3.81	<0.01	−0.12	−0.04
OC categorical	−0.04	0.02	−1.45	0.15	−0.08	0.01
MBI-EE item scores	−0.13	0.05	−2.31	0.02	−0.23	−0.02
MBI-DP item scores	0.02	0.05	0.46	0.64	−0.07	0.12
MBI-PA item scores	0.09	0.03	2.46	0.01	0.02	0.15
MBI-EE sum scores	−1.14	0.49	−2.31	0.02	−2.11	−0.17
MBI-DP sum scores	0.12	0.25	0.46	0.64	−0.37	0.60
MBI-PA sum scores	0.60	0.24	2.46	0.01	0.12	1.08
MBI-EE categorical	0.01	0.02	0.89	0.37	−0.02	0.05
MBI-DP categorical	0.00	0.01	0.11	0.91	−0.02	0.02
MBI-PA categorical	0.01	0.00	2.36	0.02	0.00	0.01
Work missed due overload	0.01	0.01	0.64	0.52	−0.02	0.03
Currently in psychotherapy	−0.01	0.01	−1.37	0.17	−0.02	0.00
Previously in psychotherapy	−0.01	0.02	−0.64	0.52	−0.05	0.03
Depression in medical history	−0.06	0.02	−3.23	<0.01	−0.10	−0.03
Suicide attempt	0.00	0.00	−0.96	0.34	−0.01	0.00
Medication intake	0.03	0.02	1.40	0.16	−0.01	0.07
Antidepressant intake	0.00	0.01	0.10	0.92	−0.01	0.01
Sedative intake	0.00	0.01	−0.03	0.97	−0.01	0.01
Analgesic intake	0.00	0.02	0.24	0.81	−0.03	0.04
Acute/chronic illness	0.01	0.02	0.25	0.80	−0.04	0.05

*Matching variables were sex (Men, Women), age (years), full-time contract (No, Yes, not reported); main occupation (private practice, hospital, not reported), working position (senior position, assistance position, not reported). AI, robust Abadie-Imbens standard errors with caliper of 0.15. Reading example: The average weekly worktime is significantly different between years indicating an average reduction in work-time of 1.94 h*.

#### Mediation

**Figure 2:**
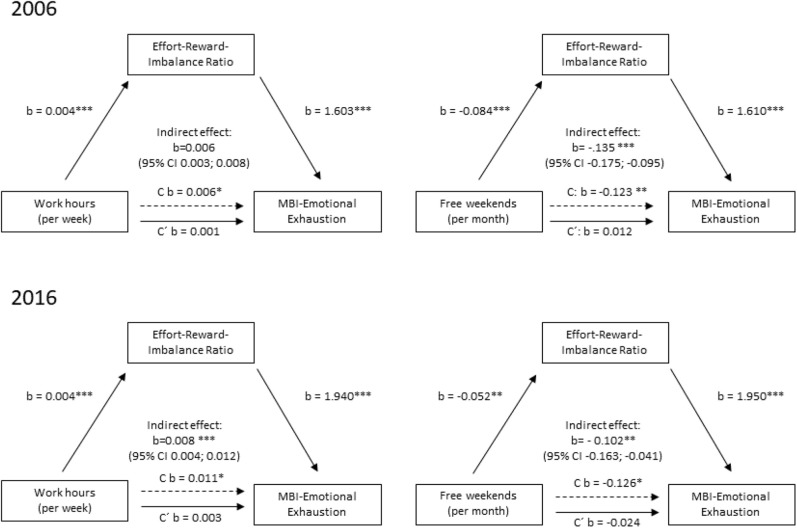
Mediation analyses of workload factors effort reward imbalance (Mediator M) and burnout (Dependent variable Y) as continuous variable per assessment year (2006 vs. 2016); all structural equation models (bootstrapped) were adjusted for age, sex, working fulltime/part-time, working in hospital/private practice and leadership/assistance position; *p <0.05, **p < 0.01, ***p < 0.001. Unstandardized coefficients are reported. C refers to the direct effect of X on Y without inclusion of the according mediator M. C′ refers to the direct effect of X on Y with inclusion of the according mediator M. For example, in 2006 per unit change in work hours per week the ERI rises by 0.004 points. Similar, per unit change in ERI, the MBI-EE scale increases on average by 1.6 scalepoints.

Figure 2 shows the unstandardized coefficients from the four different mediation models. The comparison of coefficients of 2006 and 2016 reveals a similar picture. Largest difference is seen in the association of ERI with MBI-EE [Path B].

The variable “Work hours (per week)” was significantly positively associated with ERI [Path A1] in both years. The variable “Free weekends (per month)” was significantly negatively associated with ERI [Path A1] in both years. Furthermore, ERI was significantly associated with both MBI-EE in both years [Path B]. “Work hours (per week)” was significantly positively associated with MBI-EE, [Path C1] but not when the mediator was added to the model [Path C′]. Thus, we assume a complete mediation. In addition, “Work hours (per week)” had a significant indirect and total effect in both years. “Free weekends (per month) had a significant and total effect in both years. The effect was weaker in 2016 than in 2006.

The proportion mediated is estimated as follows: “Work hours (per week)” 2006= indirect/total effect = 0.0056/0.0061 = 92%. ERI mediated to 92% the effect of the “Work hours (per week)” on MBI-EE in 2006. ERI mediated 72% of the effect of the “Work hours (per week)” on MBI-EE.

The proportion mediated “Free weekends (per month)” 2006: ERI mediates 98 % of the effect of the “Free weekends (per month)” on MBI-EE. In 2016, ERI mediated 81% of the effect of the “Free weekends (per month)” on MBI-EE.

Post estimation results indicate significant indirect effects of “Work hours (per week)” or “Free weekends (per month)” mediated through ERI in all four mediation models (Cf. Figure 2).

## Discussion

In the two cross-sectional surveys in 2006 and 2016 presented here we assessed mental health professionals, working in intensive care, with semi-structured questionnaires to obtain data on their personal life and working conditions and with standard instruments (Effort-Reward-Imbalance Questionnaire and Maslach Burnout Inventory) (35, 44) based on the literature suggesting high risk for emotional stress and affective psychopathology in this group (1, 5, 14–16). The surveys were conducted at two annual conferences. Our aim was to investigate the connections between private and professional conditions and health-related factors (professional life, stress, family, and burnout), and their changes during the intervening decade. The data show changes in working life, stress levels, and mental impairment of intensive care physicians in the decade from 2006 to 2016, and they make it possible to identify correlations with a Working Hours Act transposed into European law at the beginning of this decade.

### Private, Occupational, and Health Situation

Between 2006 and 2016 an important legislative intervention for physicians was enacted: the German Working Hours Act (27). This law classified the assessment of on-call duty by doctors as working time, so that the legally permissible working hours of physicians were reduced. Our data show that, even after adjusting for the different composition of conference attendees, weekly working hours had decreased and free weekends had increased as one would expect from the effects of the new legislation. Beside those concerning working hours and free weekends, we observed several changes in sociodemographic composition and family structure.

The approach of the gender data from 2006 to 2016 probably reflects the increase in the number of women working in the medical field (49, 50). What is noteworthy is the higher mean age and the higher percentage of the number of surveyed intensive care physicians in leadership positions in 2016 compared to 2006. This suggests a development whereby younger physicians, who tend to be residents, attend conferences less frequently than their older colleagues and colleagues in leadership positions than in the past. It is also possible that the higher percentage of women might have contributed to this finding if women attend conferences less frequently because of family commitments (51). To avoid possible confounds arising from the different composition of participants in the two cohorts, we conducted analyses where the samples were matched for these variables.

A possible reason for the significantly lower number of weekly working hours in 2016 compared to 2006 might in part be the higher number of part-time positions. However, also the number of hours worked by full-time participants decreased significantly in these 10 years. The amendment and implementation of the German Working Hours Act (27) may have played a role in this respect. The increase of free weekends without being on call between 2006 and 2016 points in the same direction. It is also possible that a changed attitude about the profession and the preparedness to take time off, especially in younger physicians, may have contributed to this development (52, 53).

What is noteworthy regarding the health-related data is that there were no significant changes in the incidence of reported acute and chronic illnesses and in the use of medication between 2006 and 2016. At the same time, the incidence of reported depressive episodes in the medical history of the physicians surveyed decreased significantly by almost 6 percentage points. However, the incidence is still relatively high at 16.5% in 2016 in comparison with the general population. The lifetime prevalence of a depressive episode in Germany is currently 11.6% (54). With regard to suicidal tendencies, the physicians we interviewed seem to be relatively unaffected. The rates of attempted suicides of the physicians surveyed (0.9 % in 2006; 0.5% in 2016) were lower than the lifetime prevalence of attempted suicides in Germany (1.7%) (55). The empirical literature indicates that the suicide rate for physicians is by 1.4-2.7 times higher than that in the general population or for members of other academic professions (33, 34). Naturally, we could only ask about suicide attempts. Interestingly, the rates of attempted suicides decreased from 2006 to 2016, even though we cannot make any statements on significance due to the small number of cases. According to the ERI model, the decline could be attributed to the decline in stress and burnout risk from 2006 to 2016 (28, 30).

There were no significant changes in reported depressive episodes in the medical history, but the percentage of those who had the diagnosis confirmed by a medical specialist increased from 2006 to 2016 by nearly 12% percentage points. This tendency gives us reason to hope that the stigmatization associated with a psychiatric diagnosis has decreased within the medical community (56), even if the difference is not statistically significant. Also, the percentage of practitioners accepting psychotherapeutic help in the past is nearly as high as the rate of those who suffered from a depressive episode in the past and did not decrease parallel to the frequency of reporting depression in the medical history. This may be indicative of a beginning change in the acceptance of psychiatric help among intensive care physicians. This is remarkable, as doctors often find it difficult to switch to the role of the patient, and mental illness is stigmatized even among physicians (57, 58). Notwithstanding these improvements, the propensity to avail oneself of psychiatric or psychotherapeutic treatment in the face of the high self-reported morbidity remains remarkably low.

This is also reflected in the sick leave figures, which did not differ significantly from 2006 to 2016, but appear low compared to those for depressive episodes in the past and acute/chronic disease at the time the survey was conducted. Possibly this points to a culture in medicine of not taking time off when sick (59).

As a note of caution, it should be mentioned here that the data on depression in the medical history and attempted suicides are based on a retrospective self-assessment. It is possible that intensive care physicians may not be proficient in the assessment, perception, and interpretation of psychiatric symptoms. Nevertheless, our data are consistent with previous reports of severe stress in intensive care physicians caused by the specific challenges in this area of work (5, 60).

### Effort-Reward-Imbalance, Overcommitment, and Burnout

The main findings of our study concern burnout risk and occupational stress. The ERI model proposed by Siegrist measures effort/reward crises at the workplace. If an Effort-Reward-Imbalance persists, the individuals in question may develop psychological and physiological stress symptoms that may be harmful in the long run (28, 37). The ERI model also takes the individual overcommitment into consideration, which, if excessive, may also lead to a higher risk for illness (37).

Interestingly, the surveyed intensive care physicians report high levels of overcommitment in both years, while the Effort-Reward-Imbalances score for this group was significantly lower in 2016. This can be seen at a glance in the comparison of dichotomous variables (Figure 1). A possible explanation is that not the intrinsic factor “overcommitment,” that obviously remained unchanged high over the decade, but both, the number of hours worked per week and the frequency of on-call weekends, which decreased significantly between 2006 and 2016, have reduced the experience of work-related stress (Effort-Reward-Imbalance). The correlation analysis and the computed mediation model confirm this assumption.

In the domains “Emotional Exhaustion” and “Personal Accomplishment” of the Maslach Burnout Inventory the continuous variables show a significant difference between 2006 and 2016, with lower scores in 2016. An explanation for this improvement in burnout risk could be the reduced occupational stress respectively the reduced Effort-Reward-Imbalances. This in turn would be consistent with Siegrist's model, which suggests that Effort-Reward-Imbalances lead to psychological stress symptoms and health impairments (28). It is also consistent with Maslach and Jacksons Burnout model. They see working conditions and organizational structures as the main cause of burnout symptoms, and interpret burnout as a mismatch between a human being and working conditions (42).

In spite of the improvement in terms of Effort-Reward-Imbalances and burnout risk since 2006, the figures remain relatively high in comparison to the general population.

In this study 8.2% of the physicians reached the cutoff for an acute burnout risk, the item means for 2016 were 3.0 (EE), 2.48 (DP), and 4.74 (PA) and thus significantly lower than the values found by Wang et al. (43) in a cohort of 475 physicians in China. The authors report item means of 3.49 for EE, of 2.27 for DP and of 5.12 for PA. The data are only comparable to a limited extent, since the authors used a 19-item revised Chinese version of the MBI with a rating of items from 1 to 7 and a cut-off of 4.5 (43). The “Study on the Health of Adults in Germany” (61) reports a burnout prevalence of 4.2% for German employees. A comparison of burnout in US physicians and in the general population in 2012, using a 22-item version of the MBI, revealed a burnout sum score of 21.0 for “Emotional Exhaustion,” 5.0 for depersonalization and 42.0 for personal accomplishment in the physician's sample and significantly higher risk for “Emotional Exhaustion,” “Depersonalization” and overall burnout compared to the matched population sample (3). In a survey among members of the State Chamber of Physicians of Saxony, Germany, similar sum scores for “Emotional Exhaustion” (21.3), higher sum scores for Depersonalization (9.9) and lower scores for Personal Accomplishment (36.3) were found (7). Our findings indicate an even less favorable situation in the 2016 sample (EE: 27.0; DP: 12.4, PA: 33.2), although Burnout scores showed a positive trend since 2006 to the effect that MBI-EE item means and sum scores decreased significantly. The scores for MBI-DP and MBI-PE also decreased, but this change was not statistically significant. However, the comparison of our data with the data of Wang et al. (43), Shanafeldt et al. (3), and Pantenburg et al. (7) is only possible to a limited extent, since we used a 21-item version of the MBI (38) and coded the items with 1-6, while our colleagues used other versions of the MBI and coded the items differently. This could explain the higher values in our study.

The evaluation of data from the Second German Sociomedical Panel of Employees identified an Effort-Reward-Imbalance ratio > 1 among 25.9% employees in the general population (62). Larisch et al. (63) found an Effort-Reward-Imbalance ratio > 1 in 16.1% among 315 employees of a metropolitan transport company. Pantenburg et al. (7) reported an averaged Effort-Reward-Imbalance-Ratio of 1.3 and mean values for effort, reward, and overcommitment of 17.9, 31.2, and 14.2, which are all well above the values in our sample in 2016.

### Correlations Between Occupational and Private Factors and Effort-Reward-Imbalance and Burnout

In our correlation analyses we looked at relations between gender, professional position, weekly working hours, free weekends, partnership, and children with Effort-Reward-Imbalance (ER-Ratio > 1), overcommitment and the dimension “Emotional Exhaustion” of burnout syndrome. Depression and suicide attempts in medical history were excluded on the one hand because we were interested in the current situation at the time of the survey. On the other hand, the data collected by standardized instruments are more objectifiable, as non-standardized requested data on the prehistory. Particularly in questions of depression and suicide tendencies, it must be considered that in the context of a congress these topics may be avoided and there is a tendency to trivialize earlier difficult phases of life.

We decided to consider only the dimension “Emotional Exhaustion,” since Maslach et al. (42) described this dimension to be the central element of burnout, and the most obvious manifestation of this complex construct (42). In addition, the data from Wang et al. (43) show that the dimension “Emotional Exhaustion” in particular is significantly related to long working hours, high job effort, and low reward. It has also been shown that “Emotional Exhaustion” is particularly influenced by Effort-Reward-Imbalance with effects to quality of care [e.g., Weigl et al. (36)].

The correlation analysis revealed correlations between weekly working hours and free weekends and OC, ERI, and MBI-EE. We tried to clarify by means of a mediation analysis whether these effects are direct or whether the influence of working time on burnout is mediated by ERI. Overcommitment was left out, since OC is the intrinsic (and relatively stable) component of the ERI model and, at high levels, leads to the stress experience being intensified by gratification crises (28, 37). Since this effect is already established in the model and the OC values had changed little between 2006 and 2016, we did not consider OC in a mediation analysis.

The mediation analyses showed a complete mediation of the relationship between working time and “Emotional Exhaustion” as a core dimension of burnout through Effort-Reward-Imbalance. We could determine this for the data from 2006 and also for the data from 2016, although in these 10 years Effort-Reward-Imbalance has clearly decreased, as well as “Emotional Exhaustion.” And we were able to determine this mediation effect both for the weekly working time and for the number of free weekends per month. In particular, the indirect effect of free weekends was highly significant. This seems essential to us because we suspect that there is a difference in the recovery effect between e.g. half an hour less work per day and one free weekend day more per month.

According to the correlation and mediation analysis and the decreasing rates in both, weekly working hours/free weekends and Effort-Reward-Imbalance, from 2006 to 2016, there seems to be a solution to the problem of high occupational stress and burnout risk: fewer working hours per week and more free weekends. This solution poses considerable difficulties. Reduced working hours may mean that more physicians need to be hired. This means additional financial expenditure for the employers, additional financial expenditure for the federal state which has to train more physicians and restructuring and additional expenditure at the medical faculties. But it can also mean changing processes. Away from excessive bureaucratic tasks, toward more patient centricity with more flexibility and room for maneuver, as Smaggus' work suggests (64).

## Strengths and Limitations

One of the strengths of our study is that it addresses a very topical issue. The methodological strengths of our study lie in the recording of occupational stress and mental impairment using established models of work stress and burnout (32, 42). This enables a theory-based interpretation and points the way to appropriate interventions. Also, we were able to collect data from a large sample of physicians working in different settings and areas of intensive care medicine, and we achieved relatively high response rates, which increases the informative value of our data. A further strength lies in the prospective investigation of the effects of working conditions of physicians over a long follow up period.

In addition, some limitations of our study are noteworthy.

First of all, the samples comprise of two independent cross-sectional surveys, which limits statements about individual change over time.

Our study aims to investigate the effect of statutory working time regulation on indicators of stress (ERI and Overcommitment) and burnout. The temporal relationship between working time and stressful life or burnout has already been established by longitudinal studies in physicians (18, 20). Therefore, between the present two cross-sectional samples we assume a similar temporal relationship.

The conference where the data were obtained are the largest conferences for intensive care medicine in Germany. In 2016 about 6000 intensive care physicians participated (38). The conference offers a scientific program and training opportunities. It is aimed at both university and non-university intensive care physicians, physicians in clinics and practices. The survey conducted at these conferences enabled us to cover the range of intensive care physicians as well as possible, thus increasing the representativeness.

Yet, we collected the data with the same methods (paper pencil questionnaires) from the same target group (physicians) and at the same conference.

The studies were carried out at conferences, which means certain selection of the participants. The advantage of achieving data on a conference is a good response rate, which we probably got by the direct and personal information of the participants.

At the same time, we cannot rule out an overlap of participants, who took part on both conferences.

We discovered a small reduction of working hours in the period of 10 years. This could be a result of a change in the law, but also many other aspects have changed in 10 years that we have not recorded or could not record (i.e., increase in the proportion working part time).

The studies were carried out at intensive care conferences in Hamburg, which did not prevent a certain selection. The intuitive assumption that acutely or severely stressed and exhausted colleagues are less likely to attend a conference would mean that the study underestimates the prevalence of Effort-Reward-Imbalance and burnout. Another point is the congress venue. Hamburg is an attractive cosmopolitan city that perhaps attracts a different sample than a smaller or provincial congress venue.

For the survey at congresses, we decided with the aim of achieving a good response rate, which we wanted to achieve through the large number of participants at the congress and the direct and personal information of the participants. The return rate was then actually higher than typically expected when surveys are mailed or emailed (65).

The data collected were determined by self-disclosure, which must also be viewed critically, despite anonymous submission of the questionnaires. It is conceivable that statements on depression and suicide could be avoided in the context of a conference. In this case, we would have expected no statements to be made on these issues. As we had little missing information in questions on these topics, we assume that this was not too much of an obstacle. A fundamental tendency toward dissimulation or aggravation of symptoms and medical history, for whatever reason, cannot be recorded with questionnaires, but must be considered when collecting data using this method.

The study was conducted with intensive care physicians, which allows statements on this group, but the transferability of the results to other specialist groups must be viewed critically. Another limitation of our study is that we collected data from random samples of physicians working in Germany. The extent to which our data can be transferred to other countries is unclear.

Methodologically, it must also be considered with caution that we relate the working time surveyed to occupational stress, measured with the Effort-Reward-Questionnaire, whereby the ERI questionnaire also asks about the burden of working time in one item. Yet, we gave priority to maintain the validated instrument.

For physicians from other European countries, the data could be of interest and informative value as they show changes in working hours, stress and burnout after the implementation of European labor law. This could have implications for interventions in clinics in other European countries.

Despite the limitations our conclusions provide valuable insights into the mechanisms of how the balance between effort and reward for intensive care physicians can be maintained and positively influenced. The data on the correlations between weekly working hours, free weekends and ERI and psychological stress in the form of burnout indicate that this is a worthwhile endeavor.

In summary, our study constitutes the first large questionnaire survey on occupational and private life stress factors, burnout and health in physicians working in intensive care after a legislative intervention. Our data show positive changes in the professional and private situation as well as the emotional well-being of intensive care physicians, but there is stillroom for further improvements. The influence of the number of hours worked per week and the number of on-call weekends on the ERI illustrates the need for preventive interventions, specifically designed for physicians working in intensive care. These results should be further substantiated by prospective studies. Still, it should be possible to reduce professional stress by taking some action regarding the number of hours worked, which seems reasonable in a specialty that is characterized by high emotional stress, and the intensive and constant confrontation with “end of life” issues. Taking into consideration the tendency to overcommit, this should be supplemented by individual preventives measures as well as easily accessible therapeutic support, customized for the needs and fears of the physicians working in intensive care.

## Data Availability Statement

The datasets analyzed in this article are not publicly available because the participants were not informed that the data would be publicly available and therefore could not agree to this. Requests to access the datasets should be directed to Petra Beschoner, petra.beschoner@uni-ulm.de.

## Ethics Statement

The studies involving human participants were reviewed and approved by Ethikkommission Universität Ulm, Ulm, Germany. Written informed consent for participation was not required for this study in accordance with the national legislation and the institutional requirements.

## Author Contributions

PB, LS, MB, CS-L and JW: conceptualization. CS-L, LJ-B, JW, and MJ: statistical analysis. MB and CS-L: funding acquisition. PB, LS, and MJ: methodology. PB and LS: writing-original draft preparation.

### Conflict of Interest

The authors declare that the research was conducted in the absence of any commercial or financial relationships that could be construed as a potential conflict of interest.
